# Supplementary material for: High-resolution light field prints by nanoscale 3D printing
Source: Nat Commun. 2021 Jun 17;12:3728. doi: 10.1038/s41467-021-23964-6 (PMC8211842; doi:10.1038/s41467-021-23964-6)
Supplement: Supplementary file 1 — Supplementary Information [file 41467_2021_23964_MOESM1_ESM.pdf]

Supplementary information for High-resolution light field prints by nanoscale 3D printing

Authors: John You En Chan [1], Qifeng Ruan [1 *], Menghua Jiang [2], Hongtao Wang [1], Hao Wang [1], Wang Zhang [1], Cheng-Wei Qiu [2], Joel K.W. Yang [1,3 *]

[1] Engineering Product Development, Singapore University of Technology and Design, Singapore 487372.

[2] Department of Electrical and Computer Engineering, National University of Singapore, Singapore 117583.

[3] Institute of Materials Research and Engineering, Singapore 138634.

* To whom correspondence should be addressed. Email: qifeng_ruan@sutd.edu.sg; joel_yang@sutd.edu.sg

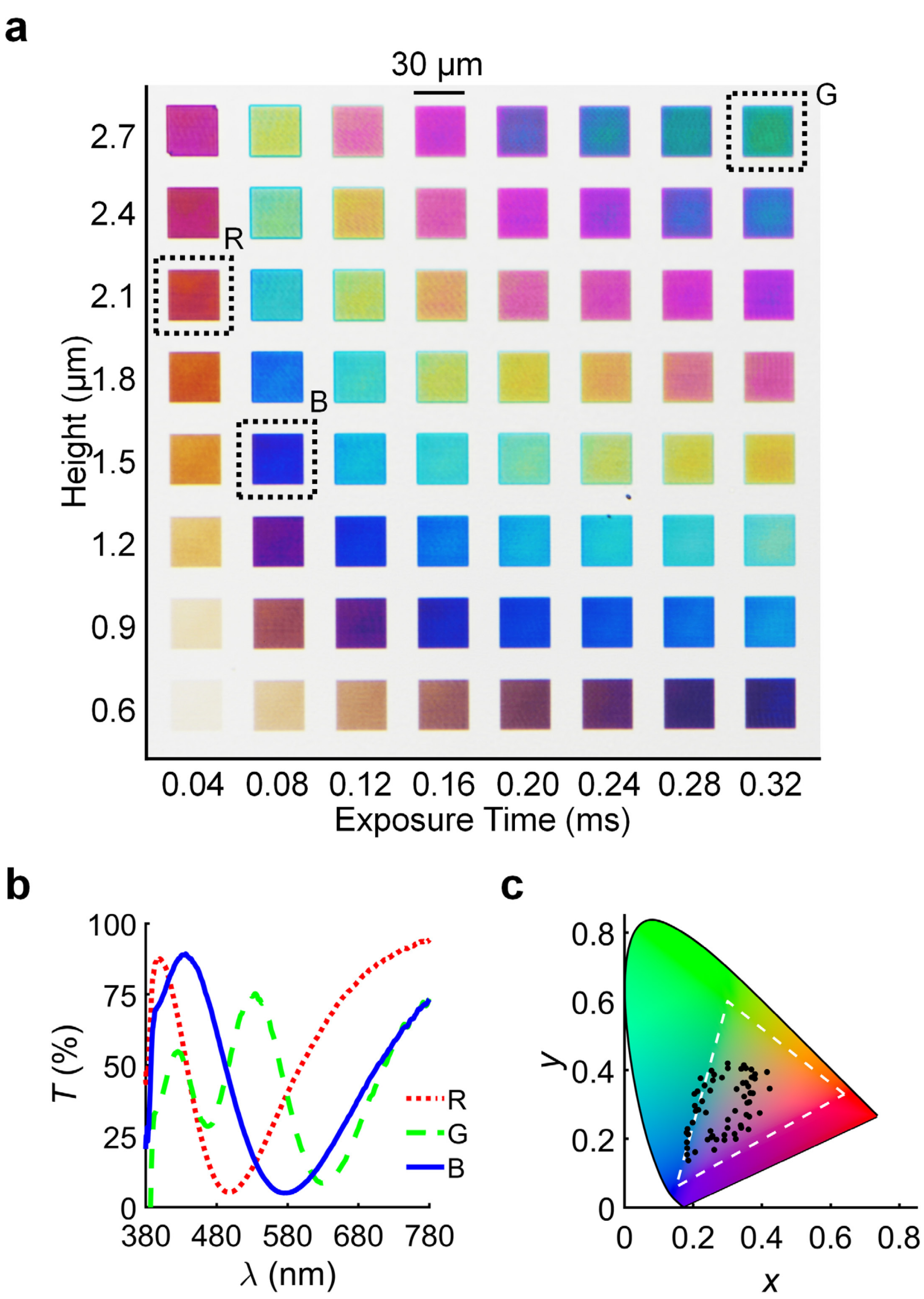

Supplementary Fig. 1. Colour measurements. (**a**) Brightfield transmission optical microscope image (taken with 10X NA 0.2 objective) showing the colour palette used to fabricate our light field print. A wide range of colour pixels was produced by varying the height of nanopillars in each pixel and the laser's exposure time per exposed voxel. The laser's power was set to 20 mW. (**b**) Experimentally measured transmittance spectra for pixels labelled R, G and B. (**c**) The CIE *xy* coordinates of all pixels were calculated and mapped onto the chromaticity diagram, for D65 illuminant and CIE 1931 2° Standard Observer. The sRGB colour space is outlined by the white dashed triangle.

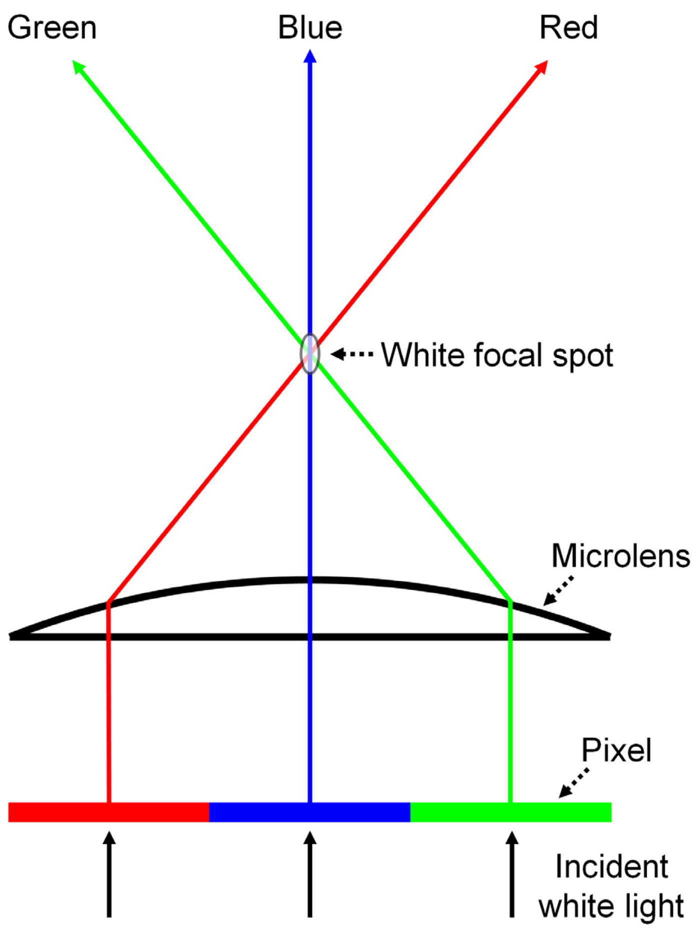

Supplementary Fig. 2. Schematic illustrating the colour-mixing effect in a display unit that comprises a microlens above a group of red, blue, and green pixels with equal areas. Normally incident light rays from the colour pixels are focused by the microlens into a white focal spot above the display unit. Beyond the focal spot, the colour of each pixel is observed in the far-field at the corresponding viewing angle.

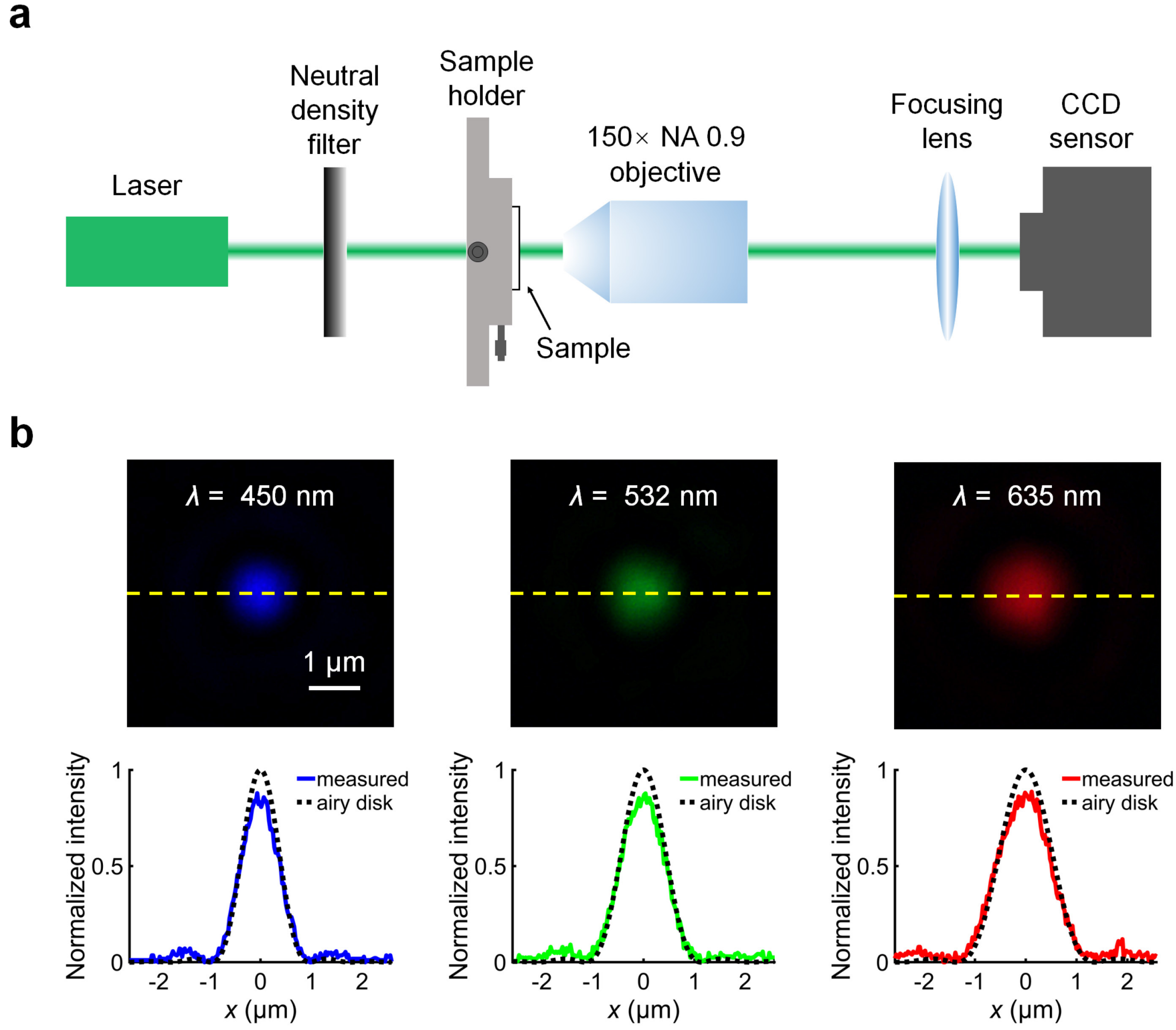

Supplementary Fig. 3. Measurement of microlens performance. (**a**) Side-view schematic of the laser setup used to capture focal spot images of a fabricated microlens. (**b**) (Top) Focal spot images for wavelengths λ = {450, 532, 635} nm. The same scale bar applies to each focal spot image. Centre horizontal slices of the focal spot images are indicated by the yellow dashed line. (Bottom) Measured intensity distributions of the horizontal slices normalized to an ideal Airy disk function for the respective wavelengths.

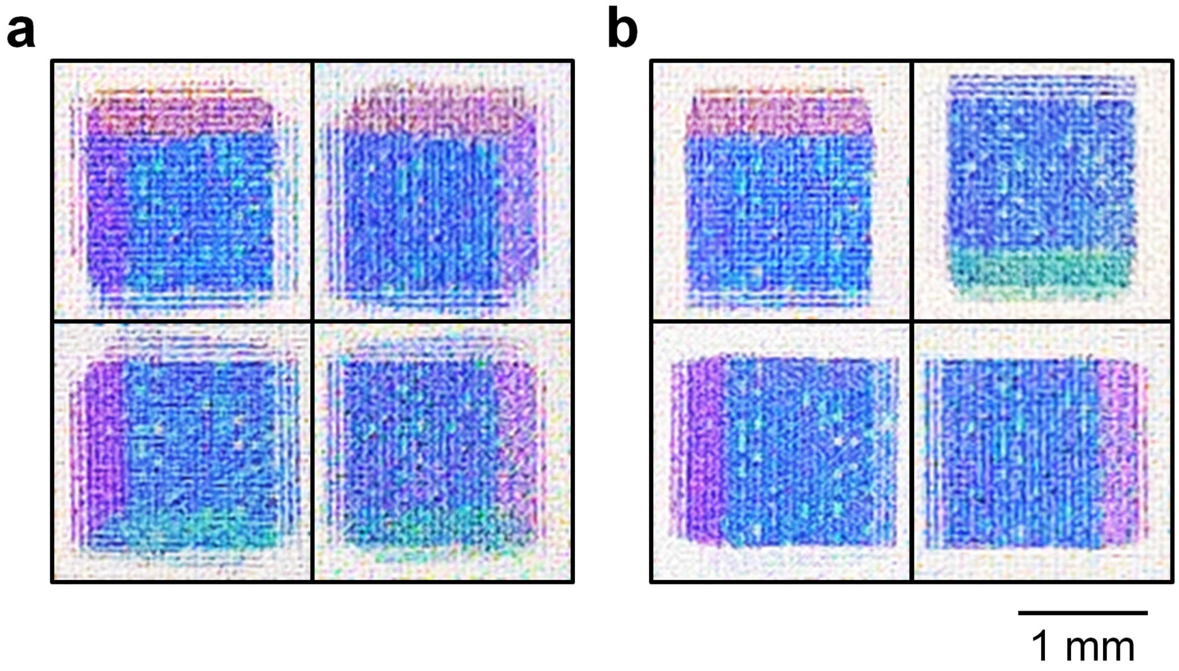

Supplementary Fig. 4. Digital camera images of crosstalk between viewpoints. (**a**) Crosstalk in the diagonal direction. (**b**) Crosstalk in the horizontal and vertical directions. The same scale bar applies to each viewpoint image.

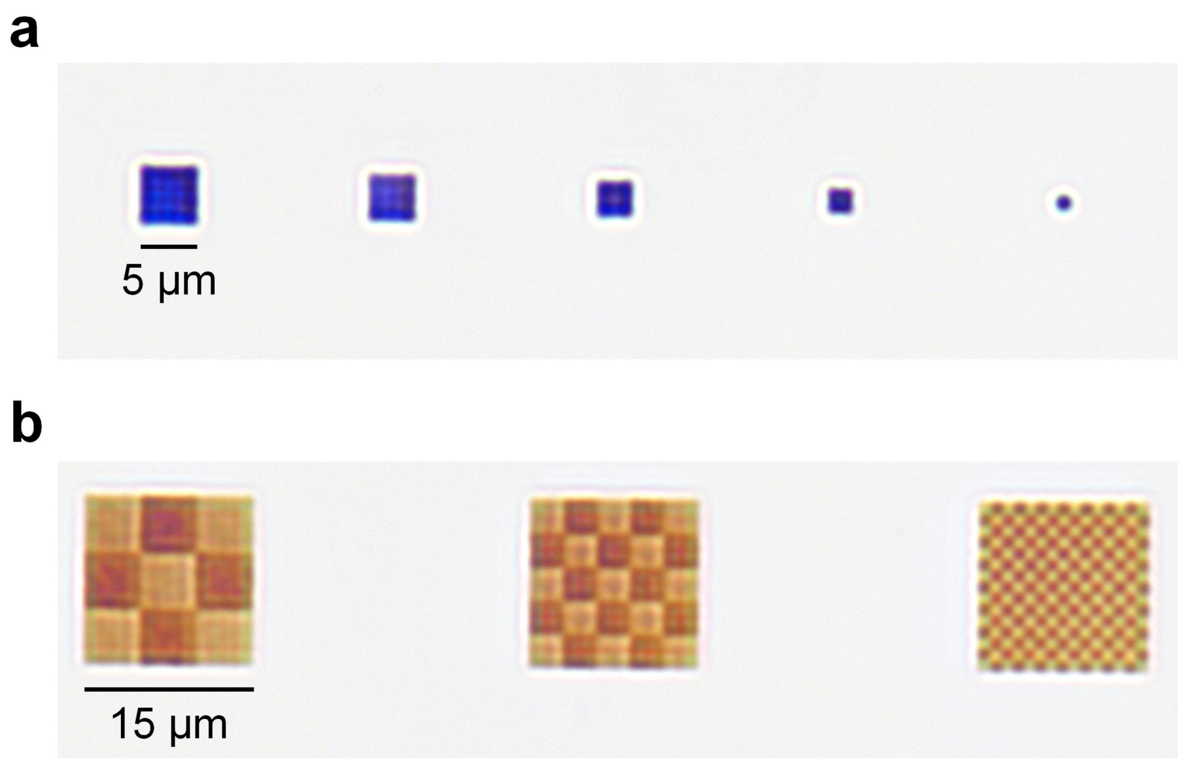

Supplementary Fig. 5. Brightfield transmission optical microscope images (taken with 50X NA 0.4 objective) showing consistent appearance of pixel colour and contrast as the pixel size is reduced. (**a**) From left to right, the pixel size is gradually reduced from 5 × 5 nanopillars to only a single nanopillar. (**b**) Chequerboard patterns of alternating light and dark colour pixels. From left to right, the pixel size in each chequerboard is reduced from 5 × 5 nanopillars to 3 × 3 nanopillars to only a single nanopillar.

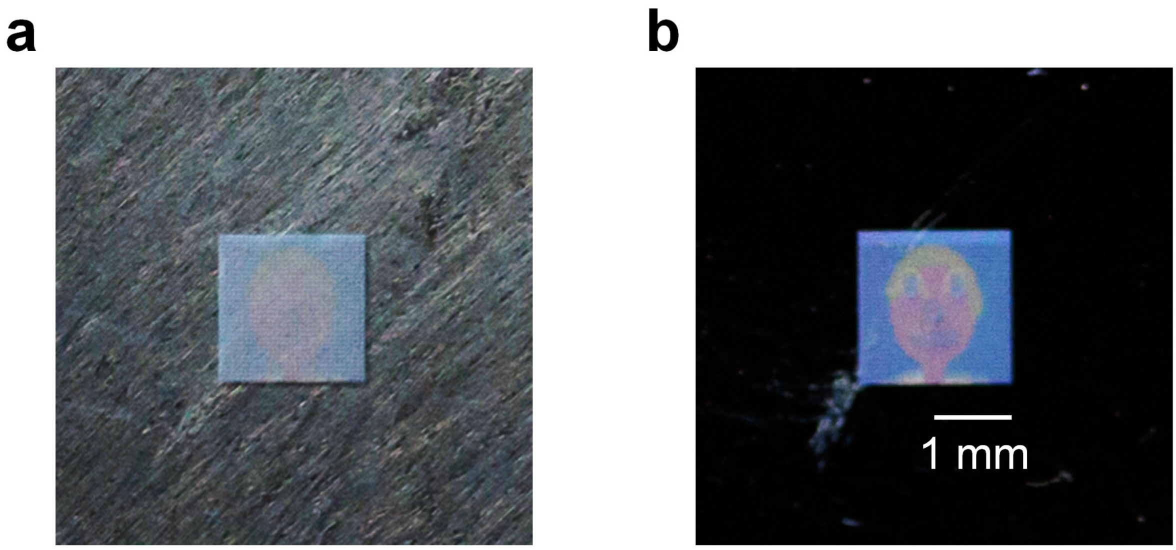

Supplementary Fig. 6. Appearance of the light field print in reflection mode, where the light source and observer are on the same side as the substrate. (**a**) A matte background was placed beneath the substrate. (**b**) An aluminium mirror was placed beneath the substrate. The same scale bar applies to both images.

Supplementary Note 1 – Geometric equations for raytracing through a lens[1]

Ignoring higher order terms, the sag *z* of a rotationally symmetric lens is expressed in Equation S1:

$$z(x,y) = \frac{x^2+y^2}{R\left(1+\sqrt{1-(1+K)\frac{x^2+y^2}{R^2}}\right)} + \cdots \tag{S1}$$

where (*x, y, z*) are the cartesian coordinates of the lens surface, *R* is the radius of curvature at the lens vertex, and *K* is the conic constant. We set *K* = 0 (spherical case) and *R* = 22 µm.

Due to the rotational symmetry of the lens, analysis of the lens profile is simplified by considering only its sagittal cross-section (*y* = 0). On this plane, the angle *σ* that the normal of the lens surface makes with the optical axis is expressed in Equation S2:

$$tan\, \sigma = \frac{\partial s}{\partial x} = \frac{x}{R(1+\varepsilon)}\left(2+\frac{(1+K)(x^2+y^2)}{\varepsilon(1+\varepsilon)R^2}\right) + \cdots \tag{S2}$$

where $\varepsilon = \sqrt{1-(1+K)\frac{x^2+y^2}{R^2}}$.

Once the normal of the lens surface is known, rays are traced through the lens by applying Snell's Law, which is expressed in Equation S3.

$$n_1 \sin\theta_1 = n_2 \sin\theta_2 \tag{S3}$$

where n_1 and n_2 are the refractive index of the first medium and second medium respectively, θ_1 is the angle of incidence, and θ_2 is the angle of refraction.

Supplementary Note 2 – Calculation of CIE XYZ and xy coordinates[2]

The CIE tristimulus values (*X*, *Y*, *Z*) are expressed in Equations S4 – S7:

$$X = k\sum_{380}^{780} T(\lambda)S(\lambda)\bar{x}(\lambda)\Delta\lambda \qquad \text{(S4)}$$

$$Y = k\sum_{380}^{780} T(\lambda)S(\lambda)\bar{y}(\lambda)\Delta\lambda \qquad \text{(S5)}$$

$$Z = k\sum_{380}^{780} T(\lambda)S(\lambda)\bar{z}(\lambda)\Delta\lambda \qquad \text{(S6)}$$

where

$$k = 100/\sum_{380}^{780} S(\lambda)\bar{y}(\lambda)\Delta\lambda \qquad \text{(S7)}$$

k is the normalizing factor, $T(\lambda)$ is the transmittance of the pixel, $S(\lambda)$ is the relative spectral power of a CIE standard illuminant, $\bar{x}(\lambda)$, $\bar{y}(\lambda)$ and $\bar{z}(\lambda)$ are the colour matching functions of a CIE standard observer, and $\Delta\lambda$ is the wavelength measurement interval.

The CIE chromaticity coordinates (*x*, *y*) are then calculated from Equations S8 – S9:

$$x = \frac{X}{X + Y + Z} \qquad \text{(S8)}$$

$$y = \frac{Y}{X + Y + Z} \qquad \text{(S9)}$$

Supplementary Note 3 – Calculation of maximum image depth[3]

In our light field print (LFP), the microlenses have centre-to-centre separation *C* and focal length *F*, whereas the pixels have pixel pitch *P*. By using similar triangles and the paraxial approximation, the maximum image depth *I* is expressed in Equation S10:

$$I = \frac{CF}{P} \tag{S10}$$

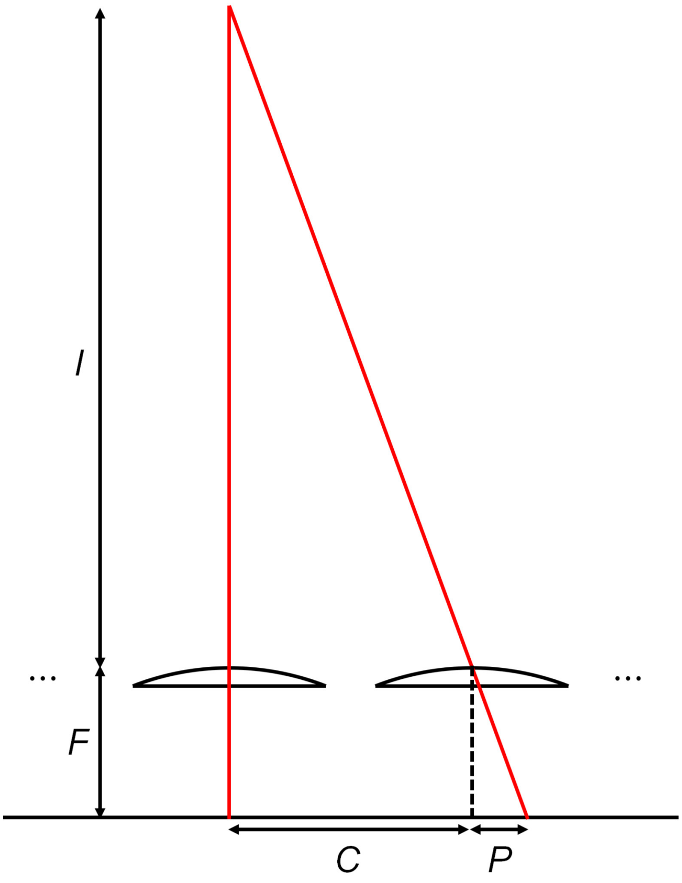

Supplementary References

1 Mikš, A., Novák, P. & Novák, J. Calculation of aberration and direction of a normal to aspherical surface. *Opt. Laser Technol.* **45**, 708-712 (2013).

2 ASTM International. Standard Practice for Computing the Colors of Objects by Using the CIE System. ASTM E308-01 (2001).

3 Kim, Y. M., Choi, K.-H. & Min, S.-W. Analysis on expressible depth range of integral imaging based on degree of voxel overlap. *Appl. Opt.* **56**, 1052-1061 (2017).
