# Supplementary material for: High-resolution light field prints by nanoscale 3D printing
Source: Nat Commun. 2021 Jun 17;12:3728. doi: 10.1038/s41467-021-23964-6 (PMC8211842; doi:10.1038/s41467-021-23964-6)
Supplement: Supplementary file 3 — Description of Additional Supplementary Files [file 41467_2021_23964_MOESM3_ESM.docx]

**Description of Additional Supplementary Files**

**Supplementary Movie 1**. Observation of the light field print while tilting it in real-time. As the light field print is tilted, changing perspectives of a cartoon face are displayed with varying colour brightness and contrast.
